# Antiviral Active Compounds Derived from Natural Sources against Herpes Simplex Viruses

**DOI:** 10.3390/v13071386

**Published:** 2021-07-16

**Authors:** Lukas van de Sand, Maren Bormann, Yasmin Schmitz, Christiane Silke Heilingloh, Oliver Witzke, Adalbert Krawczyk

**Affiliations:** 1 West German Centre of Infectious Diseases, Department of Infectious Diseases, University Hospital Essen, University of Duisburg-Essen, 45147 Essen, Germany; lukas.vandesand@uk-essen.de (L.v.d.S.); maren.bormann@uk-essen.de (M.B.); christiane.heilingloh@uk-essen.de (C.S.H.); oliver.witzke@uk-essen.de (O.W.); 2 Institute of Virology, University Hospital Essen, University of Duisburg-Essen, 45147 Essen, Germany; yasmin.schmitz@stud.uni-due.de

**Keywords:** herpes simplex viruses, natural products, antiherpetic drugs, resistance

## Abstract

Herpes simplex viruses (HSV) are ubiquitously distributed with a seroprevalence ranging up to 95% in the adult population. Refractory viral infections with herpes simplex virus type 1 (HSV-1) and type 2 (HSV-2) represent a major global health issue. In particular, the increasing occurrence of resistance to conventional antiviral drugs make the therapy of such infections even more challenging. For instance, the frequent and long-term use of acyclovir and other nucleoside analogues targeting the viral DNA-polymerase enhance the development of resistant viruses. Particularly, the incidental increase of those strains in immunocompromised patients is alarming and represent a major health concern. Alternative treatment concepts are clearly needed. Natural products such as herbal medicines showed antiherpetic activity in vitro and in vivo and proved to be an excellent source for the discovery and isolation of novel antivirals. By this means, numerous plant-derived compounds with antiviral or antimicrobial activity could be isolated. Natural medicines and their ingredients are well-tolerated and could be a good alternative for treating herpes simplex virus infections. This review provides an overview of the recent status of natural sources such as plants, bacteria, fungi, and their ingredients with antiviral activity against herpes simplex viruses. Furthermore, we highlight the most potent herbal medicines and ingredients as promising candidates for clinical investigation and give an overview about the most important drug classes along with their potential antiviral mechanisms. The content of this review is based on articles that were published between 1996 and 2021.

## 1. Introduction

Herpes simplex virus infections are considered a major public health issue worldwide. These human pathogen DNA viruses belong to the family of *Alphaherpesvirinae*. Upon primary infection, the herpes simplex viruses type 1 and 2 (HSV-1 and HSV-2) persist lifelong in the autonomic and sensory ganglia of its host. Especially HSV-1 infections are ubiquitously distributed with a seroprevalence ranging up to 95% in the adult population [1]. After reactivation, HSV may cause symptoms ranging from painful, but self-limited infections of the oral or genital mucosa to severe infections of the eye or life-threatening infections in immunocompromised hosts or newborns [2,3]. Active HSV-1 infections are usually associated with oral or facial herpes, while HSV-2 predominately causes genital infections. Reactivated HSV-2 infections often exhibit painful genital lesions providing a higher risk for other sexually transmitted diseases and invasive cervical carcinoma [4].

Although numerous vaccine candidates have been investigated in clinical trials, there is no licensed vaccine available for the prevention of HSV infections. Over the last decades, many different antiviral drugs targeting the viral DNA-polymerase were approved for the treatment of acute HSV infections. The most widely used antiviral agents against HSV are acyclovir (ACV), valacyclovir, famciclovir, cidofovir, and foscarnet. ACV and the related nucleoside analogues can successfully inhibit viral replication and thereby mediate cure from HSV-related symptoms, but the emergence of drug resistance to ACV has created a barrier for the treatment of HSV infections [5]. Moreover, it has been demonstrated that ACV therapy in HIV/HSV-co-infected patients reduces HIV serum levels and may protract the necessity of an antiretroviral therapy [6,7,8]. However, ACV may interact directly with the HIV reverse transcriptase in HIV-infected cells, which may increase the occurrence of the reverse transcriptase mutants that are associated with a reduced sensitivity of the virus to antiretroviral therapy [9,10]. Furthermore, corneal morbidity and blindness are common issues of ACV-refractory HSV infections of the cornea in industrial nations [11]. ACV-resistant infections are frequently observed in immunocompromised patients. Due to a long-term prophylactic or pre-emptive antiviral treatment in these patients, the occurrence of ACV resistance is particularly high in this group [12]. In the average adult population, the frequency of ACV-resistant HSV was determined with 0.27% (*n* = 368) [12]. In contrast, the frequency of ACV-resistant HSV was assessed with 7.03% in immunocompromised patients [12]. The highest rates of ACV resistance were reported in patients undergoing hematopoietic stem cell transplantation (14.3%), followed by HIV-infected patients (3.92%) or patients suffering from various tumor diseases (3.85%) [12]. Cross-resistance to other nucleoside analogs targeting the viral HSV thymidine kinase (TK) are frequent, since a reduced sensitivity of HSV to ACV is mostly caused by mutations in the TK gene [5,13]. However, resistance not only emerges against drugs such as famciclovir or penciclovir that target HSV-TK but also against the viral DNA-polymerase inhibitors foscarnet and cidofovir [14], the latter severely in patients undergoing stem cell transplantation [12]. DNA-polymerase inhibitors can be used for the treatment of ACV-resistant HSV infections. However, their use is limited due to possible serious side effects, especially in patients with comorbidities [5].

Clearly, there is an urgent need to explore new effective and well-tolerated approaches for the treatment of HSV infections and reactivations. Traditional herbal medicines are an abundant source of antimicrobial or antiviral active substances. Plant extracts and other natural products have been used for hundreds of years for the treatment of infectious diseases. We screened the PubMed database to find relevant articles by using the keywords natural products, medicinal plants, medicinal herbs, herbal medicine, plant oils, herpes simplex virus, herpes labialis, and herpes genitalis. Articles included in our analysis were published from 1996 to 2021. We searched for studies that described compounds that were isolated from natural sources such as plants, fungi, and other sources. We focused on well-characterized compounds with already uncovered mechanisms of how these compounds interfere with the viral replication. This strategy allows for conclusions about the potential antiviral activity of these compounds against ACV-resistant viruses. The review gives an overview of the distinct compounds isolated from plants and other natural sources and summarizes the results from in vitro and in vivo studies conducted thus far.

## 2. Antiviral Active Ingredients from Natural Sources

**Figure 1:**
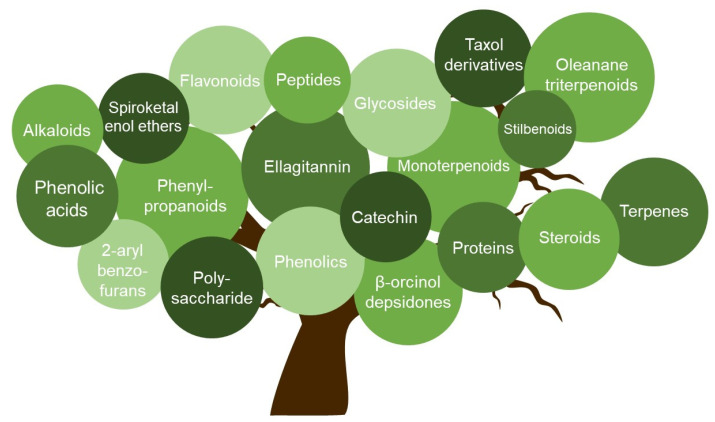
Classification of antiherpetic agents derived from natural sources. Natural sources such as plants or fungi are rich in different groups of bioactive compounds. Several of these groups (e.g., alkaloids, terpenes, polysaccharides, flavonoids, phenolic acids, and steroids) were identified as antiviral active agents against HSV-1 and HSV-2.

**Figure 2:**
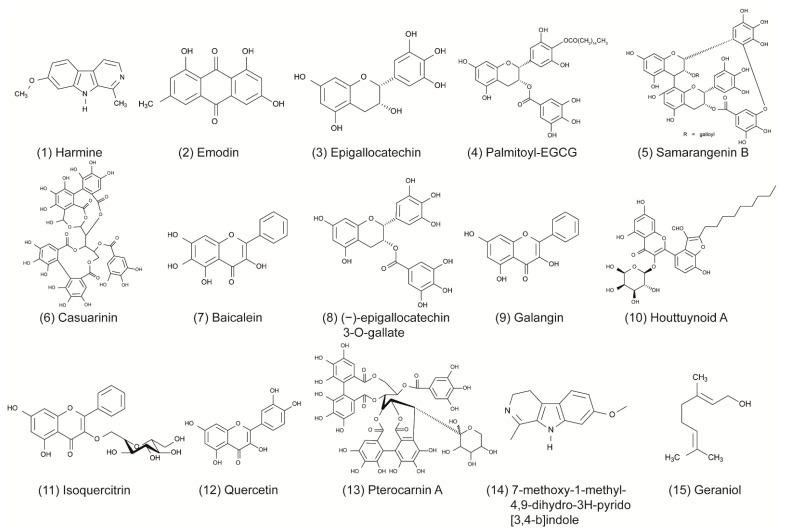

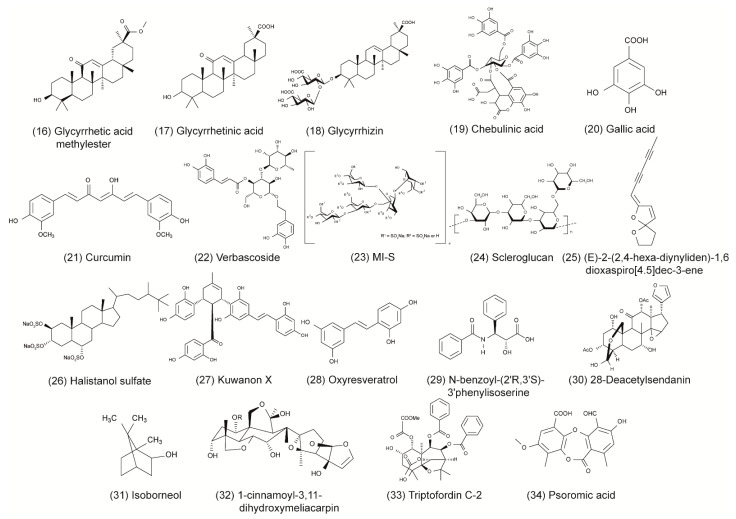
Chemical structures of distinct compounds with antiviral activity against herpes simplex viruses, which were isolated from natural sources. Enumeration is consistent with Table 1, where the characteristics of the compounds are summarized in greater detail.

Herbal medicines have been used for centuries to treat infectious diseases. Within the last decades, numerous compounds with antiviral activity against HSV and other viruses could be isolated from distinct natural sources such as plants or fungi. The antiviral active ingredients include alkaloids, terpenes, polysaccharides, flavonoids, phenolic acids, and steroids (Figure 1). The compounds inhibit the viral replication by using different mechanisms, which are summarized in Figure 2 and Table 1 and described below in more detail.

### 2.1. Inhibition of Viral Replication

**Figure 3:**
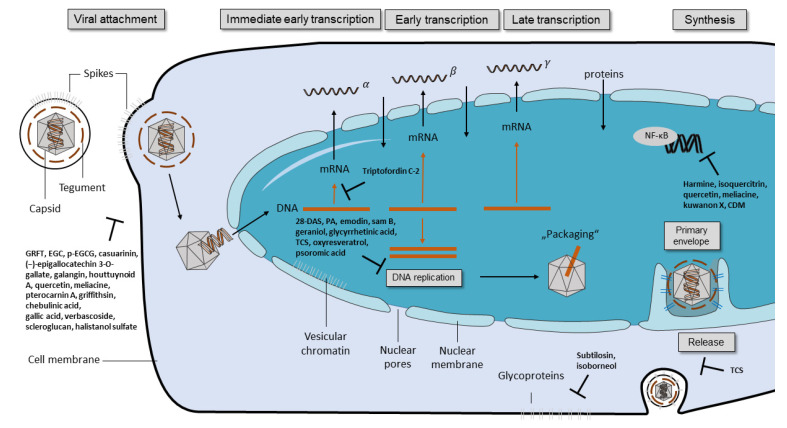
Replication cycle of herpes simplex viruses, including the targets of different natural antiviral compounds. During viral attachment, the viral glycoproteins B, D, and heterodimers of gH/gL bind to the host cell receptors. The transcription phase is divided into immediate-early, early, and late transcription where HSV genes are transcribed as α-, ß-, and γ-genes, respectively. These mRNAs are translated into immediate-early, early, and late proteins. Following DNA replication and the synthesis of the viral capsid, the primary envelopment of the capsid takes place. Subsequently, the capsids are released from the nucleus. Then, virions are released after secondary envelopment. Antiviral compounds may directly interfere with the distinct steps of the viral replication cycle or influence the cellular factors that are important for viral replication. Substances disturbing the viral replication cycle are 28-DAS, PA, emodin, sam B, geraniol, glycyrrhetinic acid, TCS, oxyresveratrol, and psoromic acid. Compounds such as harmine, isoquercitrin, quercetin, meliacine, kuwanon X, and CDM exhibit antiviral effects by addressing the primary essential cellular processes. The transcription factor NF-κB is activated by HSV-1 and HSV-2, which favors the infection. GRFT = griffithsin, EGC = epigallocatechin, *p*-EGCG = palmitoyl-EGCG, 28-DAS = 28-deacetylsendanin, PA = psoromic acid, TCS = trichosanthin, CDM = 1-cinnamoyl-3,11-dihydroxymeliacarpin.

The major targets of substances inhibiting HSV replication are the viral enzymes DNA-polymerase and TK (Figure 3). Numerous compounds affecting HSV-TK or the viral DNA-polymerase could be isolated from plants and other natural sources. These include 28-deacetylsendanin (28-DAS), psoromic acid (PA), and samarangenin B (Sam B).

28-DAS is a terpene that was isolated from *Melia azedarach*, the Persian lilac tree. Extracts from leaves have been used as antiviral agents in traditional medicine. 28-DAS showed good antiviral activity toward HSV-1 cell culture experiments. The 50% inhibitory concentration (IC_50_) of 28-DAS was determined with 1.46 µg/mL. Subsequent analysis showed that there was a transient suppression of a 44 kDa marked viral protein, which is important for viral replication. 28-DAS affects TK (p44) levels, suggesting that 28-DAS has primary, secondary, or both effects on TK [50].

PA is a β-orcinol depsidone that was isolated from *Usnea fruticose* lichens. It was uncovered to inactivate HSV-1 DNA-polymerase, which catalyzes the synthesis of DNA during HSV replication. Moreover, it was demonstrated that PA inhibited the DNA-polymerase (IC_50_ = 0.7 μM; Ki = 0.3 μM) directly without any other prior activation or modification. In comparison, ACV (IC_50_ = 0.9 μM; Ki = 0.5 μM) is initially phosphorylated by the viral TK and modified by cellular enzymes before it can be processed by the HSV-DNA-polymerase. Based on an in silico study, PA was suggested to be a competitive inhibitor of HSV-2 DNA-polymerase. The antiviral activity was evaluated at an IC_50_ value of 1.9 ± 0.42 µM (SI: >163.2) against HSV-1 and 2.7 ± 0.43 μM (SI: >114.8) against HSV-2 using a plaque reduction assay. Combination treatment of ACV and PA remarkably improved the anti-HSV efficacy at EC_50_ (50% effective concentration) values of 1.1 ± 0.41 µM for HSV-1 and 1.8 ± 0.44 µM for HSV-2. Cytotoxicity was determined with a CC_50_ (50% cytotoxic concentration) of >310 µM [56].

Sam B is a catechin purified from *Limonium sinense*. Sam B (IC_50_ HSV-1: 11.4 ± 0.9 μM) showed higher antiviral activity than ACV (IC_50_ HSV-1: 55.4 ± 5.3 μM) in vitro, whereby cytotoxic effects could be excluded (CC_50_: >100 µM) [22]. In addition, the neutralization efficacy of Sam B did not differ between pre- and co-treatment conditions, indicating that Sam B does not interfere with the viral adsorption or penetration process. This finding supports the perception that Sam B expresses its activity at the level of viral replication. Furthermore, a decreased ICP0 and ICP4 gene expression was reported. These genes play important roles regulating β and γ gene expression, which is needed for HSV-1 replication (Figure 3). Sam B disturbs DNA-polymerase transcripts, synthesis, and consequently blocks the production of gB, gC, gD, gG, and ICP5. Moreover, Sam B reduced the level of gB mRNA significantly. Kuo et al. concluded that Sam B might be more potent in the treatment of reactivations than nucleoside analogues due to the high impact on immediate-early transcripts [21].

### 2.2. Compounds Targeting Viral Glycoproteins

The process by which HSV attaches to the cell surface and enters the cell is mediated by different glycoproteins on the surface of the virus. The attachment of the virion is initiated by an interaction between glycoprotein C and heparan sulfate carbohydrates on the cell surfaces [63]. Glycoprotein D binds to cellular receptors such as Nectin-1 or HVEM (herpes virus entry mediator) and triggers the membrane fusion together with gB, gH, and gL. Drugs targeting highly conserved epitopes on viral glycoproteins could be more resistant to mutations because particular sequences seem to be crucial for viral infectivity and fitness [64]. Hereafter, the compounds griffithsin (GRFT), (−)-epigallocatechin 3-O-gallate (EGCG), and isoborneol with their different possibilities of targeting viral glycoproteins are described.

GRFT is a peptide produced by the red alge *Griffithsia*. The results of the in vitro studies indicated an effect on the viral glycoproteins B, D, and heterodimers of gH/gL. Those are indispensable for virus entry and the HSV spread directly between adjacent cells (cell-to-cell spread). However, understanding the exact mechanisms of how GRFT interferes with the viral entry and transmission is not yet completely resolved [57]. GRFT was shown to protect target cells from HSV infection when the cells were pre-treated with the compound, indicating an inhibitory effect of GRFT on viral entry. Furthermore, GRFT was effective in inhibiting viral transmission in already infected cells (post-entry conditions) at an EC_50_ of 2.3 µg/mL [57]. Moreover, GRFT showed antiviral effectivity in vivo and protected mice from genital herpes disease after an application of 20 µL 0.1% GRFT gel pre-infection [57]. GRFT neutralized the viral infection when the cells were pre-treated with concentrations of above 500 µg/mL [57]. The authors concluded that GRFT might affect the cell-to-cell transmission of the virus, thereby blocking viral infection [57].

Polyphenols isolated from tea plants such as *Camellia sinensis* from the family of *Camelliaceae* were shown to exceed antimicrobial and antiviral properties. One isolated component with anti-HSV-activity is EGCG. Isaacs et al. discussed that EGCG has the ability to interfere with the fusion process between the viral and cellular membranes by aggregating HSV glycoproteins B and D on the viral surface [25]. This finding was supported by the result that most virions pre-treated with EGCG were not infectious but appeared visually with an intact morphology [25].

Isoborneol from the *Salvia fruticosa* plant was shown to act dually as an antiviral agent against HSV-1. Firstly, the monoterpenoid alcohol inhibited HSV-1 protein glycosylation, resulting in an impairment of viral glycoprotein processing. Secondly, isoborneol directly inactivated HSV-1 at high concentrations, which was most probably by the interaction of its alcoholic moiety with lipids present on the viral envelope [51]. Infected Vero cells were treated with 0.06% isoborneol, and the viral loads decreased below the limit of analytical detection within 24 h [51].

### 2.3. Compounds Suppressing NF-κB Activity

NF-κB is a dimeric transcription factor, sequestered in the cytoplasm and, after its translocation into the nucleus, it mediates apoptotic, immune, or inflammatory responses. It was shown that NF-κB activation increases the efficiency of progeny HSV replication. In the early stages of HSV infection, gD–receptor interaction results in NF-κB nuclear translocation (early phase of infection) [65]. Furthermore, RNA-activated protein kinase also activates this process by the degradation of IκB in a later phase of infection [65]. Activated nuclear NF-κB participates in the synthesis of anti-apoptotic factors, which prevent a premature infection-induced cell death [66]. Based on these findings, compounds inhibiting NF-κB activation or translocation may disturb viral replication through programmed cell death (Figure 3).

Kuwanon X is a stilbene derivative from the mulberry tree (*Morus alba* L.). In addition to inhibitory effects against viral penetration and IE gene expression, kuwanon X therapy leads to NF-κB inactivation. Kuwanon X showed to be more efficient in simultaneous (IC_50_ HSV-1: 2.2 μg/mL) than in post-penetration treatment (IC_50_ HSV-1: 3.0 μg/mL). This difference might be caused by the blocking of viral adsorption and penetration by kuwanon X. Additionally, kuwanon X was shown to inhibit IE gene expression, which led to a reduced ICP4 and ICP27 protein synthesis. Those proteins are crucial for NF-κB nuclear translocation. The missing translocation results in an earlier programmed cell death [46].

*Melia azedarach*, more commonly known as Persian lilac tree or Chinaberry, contains the tetranortriterpenoid 1-cinnamoyl-3,11-dihydroxymeliacarpin (CDM) targeting the NF-κB nuclear translocation. CDM showed inhibitory effects against HSV-1 with an EC_50_ value of 0.78 µM. Cytotoxicity could not be determined up to a concentration of 100 µM on HCLE cells. The strong antiviral activity of CDM can be explained by multiple mechanisms. HSV glycoproteins B, C, and D are arrested into the Golgi complex by CDM causing an antiviral effect that is resilient against viral mutations. In addition, CDM blocks NF-κB translocation in conjunctival cells infected with HSV-1. Although it was not possible to identify the specific target in this pathway, an accumulation of p65 in the cytoplasm could be observed after CDM therapy. It was assumed that p65 is related to the downregulation of either ubiquitination processes or IK kinase complex, which are both possible causes of a NF-κB retention [52,53,54].

Quercetin and isoquercitrin are two major compounds of *Houttuynia cordata*, which were shown to suppress HSV replication by various mechanisms. Quercetin showed neutralizing activity at an EC_50_ value of 22.6 ± 4.2 µg/mL against HSV-1 and 86.7 ± 7.4 µg/mL HSV-2 and isoquercitrin at among 0.42 µg/mL against HSV-1. Both compounds are characterized by a similar chemical structure. They effectively locked HSV-induced NF-κB activation and NF-κB regulated IE genes transcription during the early phase of infection. Especially the ICP0 promoter was hindered by the downregulated NF-κB activation. ICP0 is meaningful for E and L gene expression. Quercetin and isoquercitrin blocked HSV-related NF-κB activation in the anti-apoptotic pathway in the late phase of infection [27]. However, only quercetin blocked the viral cell-attachment and inhibited plaque formation in pre-treated cell culture [27,67].

### 2.4. Compounds Affecting Viral Replication by Other Mechanisms

Another promising substance with antiviral activity against HSV is glycyrrhizin (Figure 3). Mainly found in the root of the licorice plant *Glycyrrhiza glabra*, it targets multiple viruses such as HSV, hepatitis B and C, HIV, and coronaviruses. The anti-HSV-1 activity was determined at an IC50 value of 225 ± 24.1 µM. The anti-inflammatory and immunoregulative properties of glycyrrhizin have been previously reported [34]. Glycyrrhizin inhibits HSV-1 entry into the cells via targeting adhesion molecules on the surface of the target cells [33,34].

Trichosanthin (TCS) is a protein derived from the roots of *Trichosanthes kirilowii* and known for its antiviral activity against HSV-1 and other viruses such as HIV-1. TCS neutralized HSV-1 at an EC_50_ of 38.4 µg/mL (subtoxic concentration). The drug synergy of combinatory treatment with TCS and ACV was observed, as the combination of TCS and small amounts of ACV showed a hundredfold higher neutralizing efficacy in vitro than TCS treatment alone [60,61,62]. The authors concluded that TCS uses a different mechanism of interfering with viral replication than ACV. Time-dependent cell culture experiments revealed an inhibitory effect of TCS against HSV-1 release, which leads to the conclusion that the virion maturation (protein synthesis and/or DNA replication) was inhibited by TCS [60,61,62]. Furthermore, an increased number of apoptotic cells in TCS treated and infected cultures was observed. HSV infections generally activate p38 Mitogen-Activated Protein Kinase (MAPK) and pro-survival protein Bcl-2. The latter blocks the mitochondrial release of cytochrome c, allowing the virus to replicate more efficiently. TCS was shown to decrease MAPK and Bcl-2 activity, most probably by inhibiting a step during viral replication [60,61,62]. However, uncovering the exact mechanism of how TCS interferes with the replication of HSV needs further investigation.

### 2.5. Efficacy of Natural Compounds In Vivo

Numerous natural compounds were shown to exhibit antiviral activity against HSV-1 and HSV-2 in vitro. To investigate whether these results can be extrapolated to living organisms, in vivo studies are necessary. However, only a few natural compounds that showed antiviral activity in cell culture experiments (Table 1) were further evaluated in animal studies. These compounds are discussed in the following section and summarized in Table 2.

The natural compound emodin contained in the plant *Rheum tanguticum* belongs to the anthraquinone family. Emodin reduced the replication of HSV-1 and HSV-2 on human laryngeal carcinoma (HEp-2) cells in vitro [17]. The antiviral efficacy of emodin was further investigated in mice. BALB/c mice were intracerebrally infected with a lethal dose of HSV-1 or HSV-2. Subsequently, the infected mice were orally treated with different amounts of emodin for seven days in 8 h intervals. The mice were observed for 40 days. Emodin protected the mice from a lethal outcome of infection in a dose-dependent manner. The survival was significantly higher in mice treated with emodin at 6.7 g/kg/day comparted to untreated control, in which all the mice died [17]. Notably, the treatment with emodin was even more effective than 0.1 mg/kg/day ACV orally in terms of increasing the survival rate and mean time to death of the infected mice [17].

Another compound that showed antiviral activity against HSV-1 in vitro and in vivo is the flavonoid baicalein, which is isolated from the roots of *Scutellaria baicalensis* [24]. Oral administration of baicalein at a dose of 200 mg/kg/day for seven days significantly increased the survival rate of mice that were intranasally infected with a lethal dose of HSV-1 [24]. However, the survival rate was higher in the ACV group (50 mg/kg/day) than in the baicalein group (200 mg/kg/day). The natural compound oxyresveratrol isolated from *Artocarpus lakoocha* exhibited an antiviral effect against HSV-1 and HSV-2 in vitro by inhibiting the early and late viral genes [47,48]. However, the administration of the compound only slightly increased the survival rate of mice with a cutaneous HSV-1 infection, and there was no significant difference compared to an untreated control [47].

Some compounds were also tested for their antiviral effectivity in treating genital HSV-2 infections, including curcumin, GRFT, meliacine, 7-methoxy-1-methyl-4,9-dihydro-3H-pyrido [3,4-b]indole (HM), and MI-S [30,57,68,69,70,71]. Intravaginal administration with 100 µg curcumin six hours before vaginal infection with HSV-2 significantly increased the survival time of mice compared to the untreated control [68]. However, prophylactic treatment with curcumin could only delay the lethal outcome of infection, and all mice died after 20 days post infection. Further in vivo studies with other natural compounds including the peptide GRFT isolated from the alga *Griffithsia*, the harmaline HM from the herb *Ophiorrhiza nicobarica Balkr*, as well as the polysaccharide MI-S isolated from the fungus *Agaricus brasiliensis* showed that these compounds significantly increased the survival rate of mice intravaginally infected with HSV-2 [30,57,69]. In the experiments, MI-S and GRFT were vaginally administered before infection, and HM was orally administered after infection with HSV-2. However, depending on the dose of HM administered, the survival rate of mice treated with ACV was at least 10% higher compared to HM treatment [30]. Furthermore, the terpene meliacine, from which CDM can be obtained, was shown to protect mice from developing severe genital HSV-2 infection. In this study, 50 mg of meliacine was vaginally administered as cream twice a day for five consecutive days [70]. In a further study, topical administration of meliciacie was also effective in preventing mice with a corneal HSV-1 infection from developing severe keratitis. Only 5% of treated mice showed signs of keratitis. The untreated group reached levels from 85 to 90% [71].

## 3. Summary and Additional Comments

The current standard therapy for HSV primary infection and reactivation includes ACV and valacyclovir. However, the emergence of drug resistances limits the available treatment options [5]. Therefore, there is a clear need for the development of new effective antiviral drugs. Plants traditionally used for medical purposes are a promising source of new antiviral compounds.

In the present review, we summarized compounds isolated from plants, bacteria, and fungi with different mechanisms inhibiting HSV, including the inhibition of viral attachment, penetration, and replication. The compounds belong to different groups such as catechins, flavonoids, phenolic acids, polysaccharides, terpenes, and peptides. With six compounds, flavonoids represent the largest group.

Some of the tested compounds showed a similar or even better antiviral activity against HSV-1 than ACV in vitro, including the anthraquinone emodin, the catechin epigallocatechin (EGC), the harmaline HM, and the β-orcinol depsidone PA as well as other promising compounds (Table 1 and Figure 1) [17,18,31,35,44,56]. Interestingly, when these compounds were supplemented with geraniol, they had a similar or better antiviral activity against HSV-2 [17,18,30,32,36,56]. These candidates might be good for further evaluation in the search for alternative treatments of ACV-resistant viruses. Additional studies are needed to investigate whether these compounds maintain their antiviral activity against HSV-1 and HSV-2 clinical isolates that are resistant to ACV. To date, only a few studies have included ACV-resistant strains in their experiments [4,47,69]. In the study of Luo et al. (2020), the antiviral effect of baicalein against an ACV-resistant HSV-1 strain (HSV-1 Blue) was comparable in its effect against the HSV-1 F strain without resistance [24]. As anticipated, ACV exhibited only a weak antiviral activity against the ACV-resistant virus. However, ACV showed a stronger inhibition against the HSV-1 F strain than baicalein. In a further in vitro study, the antiviral effect of MI-S was observed against HSV-1 KOS, an ACV-resistant HSV-1 strain (HSV-1 29R), and HSV-2 333 [69]. MI-S was similarly effective against HSV-1 KOS and HSV-1 29R when the compound was administered during or after infection. ACV showed no inhibitory activity against HSV-1 KOS when applied simultaneously with the virus, but it was more effective than MI-S under post-infection treatment conditions.

Only a few of those compounds were evaluated for their efficacy against HSV infections in in vivo studies. Of these compounds, emodin exhibited promising results, as it strongly increased the survival rate of mice infected with HSV-1 and HSV-2 [17]. The compound was even more effective than ACV in increasing the survival rate and mean time to death. Furthermore, HM significantly increased the survival rate of mice infected with HSV-2 [30]. Although standard therapy with ACV was more effective, the increase of survival through the compound was still striking. The results of these studies indicate that emodin and HM are promising candidates for future clinical trials. Clearly, there is a need for more elaborate in vivo studies as well as clinical trials that compare the compounds to the standard therapy of HSV.

## 4. Conclusions

To date, numerous herbal medicines and their main ingredients were investigated for their antiviral efficacy against HSV-1 and HSV-2 in cell culture and animal models. For instance, the anthraquinone emodin derived from the plant *Rheum tanguticum* as well as the harmaline HM isolated from the herb *Ophiorrhiza nicobarica Balkr* exhibited a strong antiviral effect against HSV in vitro and in vivo, which were comparable to or even better than ACV. Clinical studies are needed to determine the efficacy of these compounds in humans. Furthermore, natural compounds might be an effective substitute for drugs such as ACV in the treatment of ACV-resistant HSV infections. Taken together, herbal medicines represent a promising source to isolate novel compounds with antiviral activity against HSV-1 and HSV-2. Numerous compounds could be isolated and pre-clinically characterized thus far. Further clinical evaluation of the agents that are most promising in animal trials may lead to the development of novel therapy options in humans.

## Figures and Tables

**Table 1:** Chemical compounds and their anti-HSV activity. CC_50_ = 50% cytotoxic concentration, EC_50_ = 50% effective concentration, IC_50_ = 50% inhibitory concentration, SI = selectivity index, HSV = herpes simplex virus, CPE = cytopathogenic effect, XTT = 2,3-bis[2-methoxy-4-nitro-5-sulphophenyl]-5-[(phenylamino)carbonyl-2H-tetrazolium hydroxide], ELISA = enzyme-linked immunosorbent assay, MAPK = mitogen-activated protein kinase, CCECs = cerebral capillary vessel endothelial cells, PMNs = polymorphonuclear leukocytes, TK = thymidine kinase.

No.	Group	Compound	Plant/Other	Assay Employed; Cell Line	CC_50_	HSV-1 EC_50_/IC_50_; SI	HSV-2 EC_50_/IC_50_; SI	Virus	Mechanism of Action/Target Structure	Ref.
1	Alkaloid	Harmine	*Peganum harmala*	CPE; Hec-1-A cells	>300 µM	4.56 µM	1.47 µM	HSV-1 F; HSV-2 G	Tyrosine phosphorylation-regulated kinase inhibitor; downregulation of cellular NF-κB and MAPK pathways.	[15,16]
2	Anthraquinone	Emodin	*Rheum tanguticum*	CPE; HEp-2 cells	>1000 µg/mL (>3.7 mM)	*n*/A	*n*/A	HSV-1 F; HSV-2 (333)	Inhibition of viral replication.	[17]
3	Catechin	Epigallocatechin (EGC)	*Thea sinensis* L.	CPE; Vero cells	500 µM	4 μM; 125	63 μM; 7.9	HSV-1 KOS; HSV-2 G	Glycoproteins B and D.	[18,19]
4		Palmitoyl-EGCG (*p*-EGCG)		Plaque assay; Vero cells	>75 µM	<50 µM	*n*/A	HSV-1 UL46	Glycoprotein D expression is reduced.	[20]
5		Samarangenin B (Sam B)	*Limonium sinense* L.		>100 μM	11.4 μM	*n*/A	HSV-1 KOS	Suppresses expression of ICP0 and ICP4 genes and viral DNA-polymerase (ß transcripts).	[21,22]
6	Ellagitannin	Casuarinin	*Terminalia arjuna Linn*	Plaque assay; Vero cells	89 ± 1 µM	*n*/A	1.5 μM; 59	HSV-2 (196)	Inhibition of viral attachment and penetration.	[23]
7	Flavonoid	Baicalein	*Scutellaria baicalensis*	Plaque assay; Vero cells	>200 µM	*n*/A	12.4 µM; >16.1	HSV-1 F	Inactivation of free viral particles and downregulating cellular NF-κB.	[24]
				HaCat cells	>200 µM	*n*/A	20.1 µM; >9.95			
8		(−)-epigallocatechin 3-O-gallate (EGCG)	*Camellia sinensis*	Titer reduction; Vero cells	*n*/A	10^2.0^/10^4.4^ reduction at 100 μM	10^4.0–5.0/^10^4.0^ reduction at 100 μM	HSV-1 F; HSV-2 (333)	Glycoproteins B and D.	[25]
9		Galangin	*Helichrysum aureonitens*	CPE; Vero cells	1000 µM	2.5 μM; 400	*n*/A	HSV-1 KOS	Inhibition of of viral adsorption.	[18]
10		Houttuynoid A	*Houttuynia cordata*	Plaque assay; Vero cells	166.36 ± 9.27 μM	23.50 ± 1.82 μM		HSV-1 F	Blocking viral attachment.	[26]
11		Isoquercitrin	*Houttuynia cordata*		>100 µg/mL (215.34 µM)	0.42 µg/mL (0.9 µM); >512.8			Downregulation of cellular NF-κB.	[27]
12		Quercetin	*Caesalpinia pulcherrima*	XTT; BCC-1/KMC cells	496.9 µg/mL (16.44 mM)	22.6 ± 4.2 µg/mL (74.78 ± 13.89 µM); 22.0	86.7 ± 7.4 µg/mL (286.86 ± 24.48); 5.7	HSV-1 KOS; HSV-2 (196)	Downregulation of cellular NF-κB and blocks viral entry (gD cellular binding site).	[27,28]
13	Glycosides	Pterocarnin A	*Pterocarya stenoptera*	XTT; Vero cells	31.7 ± 1.6 μM	*n*/A	5.4 ± 0.3 μM; 5.9	HSV-2 (196)	Inhibition of of viral attachment.	[29]
14	Harmaline	7-methoxy-1-methyl-4,9-dihydro-3H-pyrido[3,4-b]indole (HM)	*Ophiorrhiza nicobarica Balkr*	Plague assay; Vero cells	30 µg/mL (120.14 µM)	1.1 ± 0.1 µg/mL (4.41 ± 0.4 µM); 27.27	1.5 ± 0.1 µg/mL (6.01 ± 0.4 µM); 20	HSV-1 F; HSV-2 G	Suppression of viral immediate early gene expression.	[30,31]
15	Monoterpenoid	Geraniol	*Thymus bovei*	Titer reduction; Vero cells	>210 µg/mL (1361.42 µM)	*n*/A	1.92 ± 0.84 µg/mL (12.45 ± 5.45 µM); >109.38	HSV-2	In silico: Interacts with HSV-2 protease.	[32]
16	Oleanane triterpenoid	Glycyrrhetinic acid methylester	*Glycyrrhiza glabra*	Plaque assay; Vero cells	>207 µM	8.1 ± 0.2 µM/mL; >26	*n*/A	HSV-1 KOS	Inhibition of HSV-1 replication.	[33]
17		Glycyrrhetinic acid			84.0 ± 2.8 µM	21.7 ± 0.6 µM; 3.9			Induces the autophagy activator Beclin 1 → blocks HSV replication.	[33]
18		Glycyrrhizin			>608 µM	225 ± 24.1 µM/mL; >2.7			Reduces adhesion force between CCECs and PMNs.	[33,34]
19	Phenolic acid	Chebulinic acid	*Terminalia chebula*	Plaque assay; Vero cells	>200 µg/mL	17.02 ± 2.82 µM; 18.62	0.06 ± 0.002 μg/mL	HSV-1 KOS; HSV-2 G	Prevention of HSV-1 glycoprotein-mediated cell fusion events and attachment of HSV-2.	[35,36]
20		Gallic acid	*Galla*	Plaque assay; Vero cells and GMK AH1	668.7 ± 54.5 μM	57.1 ± 2.3 μM; 11.72	33.56; 64.35 μM (during; after infection)	HSV-1 KOS; HSV-2 (333)	Inhibition of ICP2, gC, gD, and VP5 expression (effects on viral attachment).	[37,38]
21	Phenolic	Curcumin	*The curry spice turmeric*	CPE; Vero cells	49.8 ± 0.4 µg/mL (135.18 ± 1.09 µM)	*n*/A	*n*/A	HSV-1 (17)	P300/CBP histone acetyltransferase.	[39,40]
22	Phenylpropanoid	Verbascoside	*Lepechinia speciosa*	Plaque assay; Vero cells	>200 µg/mL (320.21 µM)	58 µg/mL (92.86 µM); >3.4	8.9 µg/mL (14.25 µM); >22.4	HSV-1; HSV-2 (clinical isolates)	HSV-1: prevention of viral adsorption, intracellular viral inhibition; HSV-2: inhibition of attachment and penetration.	[41]
23	Polysaccharide	MI-S	*Agaricus brasiliensis*	Plaque assay; Vero cells	2415.29 ± 389.21 µg/mL (134.18 ± 21.61 µM)	1.24 ± 0.05 µg/mL (0.07 µM), 1948; 5.50 ± 0.58 µg/mL (0.31 ± 0.03 µM), 439 (during; after infection)	0.39 ± 0.17 µg/mL (0.02 ± 0.01 µM), 6193; 4.30 ± 0.36 µg/mL (0.24 ± 0.02 µM), 562 (during; after infection)	HSV-1 KOS; HSV-2 333	Inhibition of attachment, penetration and cell-to-cell spread.	[42]
24		Scleroglucan	*Scierotium glucanicum*	CPE; Vero cells	400 µg/mL (559.83 µM)	5 µg/mL (7 µM); 80	*n*/A	HSV-1 F	Glycoproteins of HSV-1 (inhibits adsorption step).	[43]
25	Spiroketal-enol ether derivative	(E)-2-(2,4-hexa-diynyliden)-1,6dioxaspiro[4.5] dec-3-ene	*Tanacetum vulgare*	Time-of-addition assay; Vero cells	>30 µg/mL (>149.83 µM)	0.146 ± 0.013 µg/mL (0.73 ± 0.06 µM); >205	0.127 ± 0.009 µg/mL (0.63 ± 0.04 µM); >236	HSV-1 (AY240815.1); HSV-2 (HM011430.1)	Suppression of viral RNA synthesis.	[44]
26	Steroid	Halistanol sulfate	*Petromica citrina*	Plaque assay; Vero cells	13.83 ± 3.75 µg/mL (20.08 ± 5.44 µM)	5.63 ± 1.3 µg/mL (8.17 ± 1.89 µM); 2.46	*n*/A	HSV-1 KOS	Inhibition of attachment and penetration. Impairs HSV-1 gD and ICP27 levels.	[45]
27	Stilbenoid and 2-arylbenzofuran	Kuwanon X	*Moru salba* L.	Plaque assay; Vero cells	80.3 ± 3.2 µg/mL (128.15 ± 5.12 µM)	2.2 ± 0.1 µg/mL (3.5 ± 0.16 µM); 37	2.5 ± 0.3 µg/mL (3.99 ± 0.48 µM); 32	HSV-1 (15577); HSV-2 (333)	Downregulation of cellular NF-κB and viral RNA/DNA synthesis.	[46]
28		Oxyresveratrol	*Artocarpus lakoocha*		>100 µM	63.5 μM	55.3 μM	HSV-1; HSV-2	Inhibition of early and late replication.	[47,48]
29	Taxol derivative	*n*-benzoyl-(2’R,3’S)-3’phenylisoserine	*Lactarius*	CPE; Vero cells	>500 µg/mL	21.7 µg/mL (76.06 µM); >23	*n*/A	HSV-1 (McIntyre)	Inhibition of HSV-1 replication (possibly related to mitotic division).	[49]
30	Terpene	28-Deacetylsendanin (28-DAS)	*Melia azedarach*	ELISA; Vero cells	>400 µg/mL (696.11 µM)	1.46 µg/mL (2.54 µM)	*n*/A	HSV-1 (McIntyre)	Reduces activity of TK.	[50]
31		Isoborneol	*Salvia fruticosa*	Plaque assay; Vero cells	*n*/A	*n*/A		HSV-1 F	Affected TK-independent glycosylation process of viral glycoproteins B and D.	[51]
32		1-cinnamoyl-3,11-dihydroxymeliacarpin (CDM)	*Melia azedarach*	Plaque assay; HCLE cells	>100 µM	0.78 µM		HSV-1 KOS	Inhibition of glycoproteins B, gC, gD intracellular trafficking and downregulates cellular NF-κB.	[52,53,54]
33		Triptofordin C-2	*Tripterygium wilfordii*	Plaque assay; HeLa cells	89 ± 9.5 µg/mL (145.76 ± 15.56 µM)	3.7 ± 0.90 µg/mL (6.06 ± 1.47 µM); 24 ± 3.2		HSV-1 HF	Suppression of viral immediate early gene expression.	[55]
34	β-orcinol depsidone	Psoromic acid	*Usnea*	Plaque assay; Vero cells	>310 µM	1.9 ± 0.42 µM; >163.2	2.7 ± 0.43 μM; 114.8	HSV-1 KOS; HSV-2 (A234)	Inhibition of HSV replication: HSV-1 DNA-polymerase in vitro; HSV-2 DNA-polymerase in silico.	[56]
35	Peptide	Griffithsin	*Griffithsia*	Plaque assay; CaSki	no cytotoxic effect	*n*/A	2.3 μg/mL (0.18 µM)	HSV-2 (333)	Inhibition of viral attachment (cell-to-cell spread).	[57]
36		Subtilosin	*Bacillus amyloliquefaciens*	Plaque assay; Vero cells	314 µg/mL (92.3 µM)	9.6 µg/mL (2.82 µM); 33	18.2 µg/mL (5.35 µM); 17.4	HSV-1 F; HSV-2 G	Late stages of the viral replicative cycle and intracellular glycoprotein transport.	[58,59]
37		Trichosanthin (TCS)	*Trichosanthes kirilowii*	ELISA; Vero cells	416.5 ± 34.5 µg/mL (15.42 ± 1.28 µM)	38.4 ± 17.5 µg/mL (1.42 ± 0.65 µM); 10.8	*n*/A	HSV-1 F	Suppression of p38 MAPK protein and Bcl-2 gene activity, replication (E and L), DNA expression and viral release.	[60,61,62]

**Table 2:** In vivo studies of anti-HSV chemical substances. P.O. = Per Os.

Group	Compound	Plant/Other	Animal	Virus	Way of Infection	Treatment; Dose	Survival under Treatment	Compared to Control/ACV	Ref.
Anthraquinone	Emodin	*Rheum tanguticum*	BALB/c mice	HSV-1F; HSV-2 (333)	Intracerebral	P.O.; 6.7 g/kg/day	HSV-1: 61.5% HSV-2: ≈70%	HSV-1: Untreated control: 0%/ACV: ≈20% (*p* < 0.01) HSV-2: Untreated control: 0%/ACV: ≈30% (*p* < 0.01)	[17]
Flavinoid	Baicalein	*Scutellaria baicalensis*	BALB/c mice	HSV-1 F	Intranasal	P.O.; 200 mg/kg/day	75%	Untreated control: 33.3% (*p* < 0.05)/ACV: ≈90%	[24]
Harmaline	7-methoxy-1-methyl-4,9-dihydro-3H-pyrido[3,4-b]indole	*Ophiorrhiza nicobarica Balkr*	BALB/c mice (female and male)	HSV-2 G	Genital	P.O.; 0.25 and 0.5 mg/kg 6 h after infection	0.25 mg/kg: 45% 0.5 mg/kg: 70%	Untreated control: 5% (*p* < 0.05)/ACV: 80%	[30]
Peptide	Griffithsin (GRFT)	*Griffithsia*	BALB/c mice (female)	HSV-2	Intravaginal	Intravaginal topically; 20 µL 0.1% GRFT gel pre-infection	≈80%	Untreated control: ≈20% (*p* < 0.05)	[57]
Phenolics	Curcumin	*The curry spice turmeric*	BALB/c mice (female)	HSV-2 (333)	Intravaginal	Intravaginal topically; 100 µg 6 h pre-infection	0%	0%	[68]
Polysaccharids	MI-S	*Agaricus brasiliensis*	BALB/c mice (female)	HSV-2 (333)	Intravaginal	Intravaginal topically; 20 mg/mL 20 min pre-infection	60%	Untreated control: 0% (*p* < 0.0001)	[69]
Stilbenoids and 2-arylbenzofurans	Oxyresveratrol	*Artocarpus lakoocha*	BALB/c mice (female)	HSV-1 (7401H)	Cutaneous	P.O.; 500 mg/kg 8 h pre-infection and 3x daily for 7 days after infection	25%	Untreated control: 0% (*n*.s.)/ACV: 100%	[47]
Terpene	Meliacine (CDM)	*Melia azedarach* L.	BALB/c mice (female)	HSV-2 MS and G	Intravaginal	Intravaginal topically; 1 mg 2x daily for 5 days	HSV-2 MS: 20% HSV-2 G: 86%	Untreated control: HSV-2 MS: 0%; HSV-2 G: 42%	[70]
			BALB/c mice (female and male)	HSV-1 (KOS)	Corneal	Corneal topically; 3x daily 1 day pre-infection and for 3 days after infection	Development of keratitis: 5%	Untreated control: 90% (*p* < 0.001)	[71]
